# Maternal Race and Stillbirth: Cohort Study and Systematic Review with Meta-Analysis

**DOI:** 10.3390/jcm11123452

**Published:** 2022-06-15

**Authors:** Anastasija Arechvo, Despoina A. Nikolaidi, María M. Gil, Valeria Rolle, Argyro Syngelaki, Ranjit Akolekar, Kypros H. Nicolaides

**Affiliations:** 1 Harris Birthright Research Centre of Fetal Medicine, King’s College Hospital, London SE5 8BB, UK; mmar1984@gmail.com (M.M.G.); argyro.syngelaki@nhs.net (A.S.); kypros@fetalmedicine.com (K.H.N.); 2 Department of Obstetrics and Gynecology, Institute of Clinical Sciences Lund, Lund University, 22100 Lund, Sweden; 3 GKT School of Medical Education, King’s College London, London WC2R 2LS, UK; despoina.nikolaidi@kcl.ac.uk; 4 Department of Obstetrics and Gynecology, Hospital Universitario de Torrejón, 28850 Torrejón de Ardoz, Spain; 5 School of Medicine, Universidad Francisco de Vitoria (UFV), 28223 Madrid, Spain; 6 Bioestatistics and Epidemiology Platform at Instituto de Investigación Sanitaria del Principado de Asturias, 33011 Oviedo, Spain; avleria77@gmail.com; 7 Fetal Medicine Unit, Medway Maritime Hospital, Gillingham ME7 5NY, UK; ranjit.akolekar@nhs.net; 8 Institute of Medical Sciences, Canterbury Christ Church University, Chatham ME4 4UF, UK

**Keywords:** stillbirth, race, screening, pregnancy complications, singleton pregnancies

## Abstract

Accurate identification of independent predictors of stillbirth is needed to define preventive strategies. We aim to examine the independent contribution of maternal race in the risk of stillbirth after adjusting for maternal characteristics and medical history. There are two components to the study: first, prospective screening in 168,966 women with singleton pregnancies coordinated by the Fetal Medicine Foundation (FMF) and second, a systematic review and meta-analysis of studies reporting on race and stillbirth. In the FMF study, logistic regression analysis found that in black women, the risk of stillbirth, after adjustment for confounders, was higher than in white women (odds ratio 1.78, 95% confidence interval 1.50 to 2.11). The risk for other racial groups was not significantly different. The literature search identified 20 studies that provided data on over 6,500,000 pregnancies, but only 10 studies provided risks adjusted for some maternal characteristics; consequently, the majority of these studies did not provide accurate contribution of different racial groups to the prediction of stillbirth. It is concluded that in women of black origin, the risk of stillbirth, after adjustment for confounders, is about twofold higher than in white women. Consequently, closer surveillance should be granted for these women.

## 1. Introduction

Globally, an estimated two million babies are stillborn every year, and the rate of stillbirth is a sensitive marker of the quality of care around pregnancy and birth; the reported rates vary from 22.8 stillbirths per 1000 total births in west and central Africa to 2.9 in western Europe [1]. Studies from countries with populations that are of predominantly White race have consistently reported that in minority groups, such as women of Black race, the incidence of stillbirth is increased [2,3,4,5,6,7,8,9,10,11,12,13,14,15,16,17,18,19,20]. Two studies reported that the rate of stillbirth in South Asian women is higher than in white women [12,18], but in two other studies there was no statistically significant difference between the two racial groups [16,19]. One study reported that the rate of stillbirth in East Asian women is lower than in White women [19], but in another study there was no statistically significant difference between the two groups [16]. However, in most of these studies the observed relative incidence of stillbirth was not adjusted for confounding factors in maternal characteristics and medical history. In a previous study of 113,415 singleton pregnancies, we adjusted for confounding variables and reported that in women of Black race, but not in South or East Asians, the incidence of stillbirth was higher than in White women [21].

The aims of our screening study of 168,966 singleton pregnancies are, first, to examine the association between maternal race and stillbirth after adjustment for confounding factors in maternal characteristics and medical history, and second, to carry out a systematic review of the literature and meta-analysis of the data from independent primary studies focused on race and stillbirth.

## 2. Materials and Methods

### 2.1. Fetal Medicine Foundation Study

This was a prospective study in women with singleton pregnancies attending their first routine pregnancy hospital visit at 11 + 0 to 13 + 6 weeks of gestation at King’s College Hospital, London or Medway Maritime Hospital, Kent, England from March 2006 to November 2020. The visit included the recording of maternal demographic characteristics and medical history, measurement of maternal weight and height and ultrasound examination for the measurement of the fetal crown–rump length (CRL) to determine gestational age [22], measurement of the fetal nuchal translucency thickness as part of screening for trisomies [23], and examination of the fetal anatomy for the diagnosis of major fetal defects [24].

Participants completed a questionnaire on their age, race (White, Black, South Asian, East Asian, and mixed), method of conception (natural, assisted by in vitro fertilization or use of ovulation drugs), cigarette smoking during pregnancy, medical history of chronic hypertension, diabetes mellitus, systemic lupus erythematosus (SLE) or antiphospholipid syndrome (APS), and obstetric history that included parity (parous or nulliparous, if no previous pregnancies at ≥24 weeks’ gestation), previous pregnancy with miscarriage, previous pregnancy with stillbirth, previous pregnancy complicated by preeclampsia and previous pregnancy with delivery of small for gestational age (SGA) neonate with birth weight <10th percentile of The Fetal Medicine Foundation (FMF) fetal and neonatal population weight charts [25]. In relation to race, the patients were asked to choose one of White, Black, South Asian, East Asian, or mixed, and they were also asked to record the country of origin of each parent. The questionnaire was reviewed by a doctor together with the pregnant woman. In case of a language barrier, professional translation services were offered to the participants.

The inclusion criteria for this study were singleton pregnancies delivering a non-malformed live birth or stillbirth at ≥24 weeks’ gestation. Pregnancies resulting in a pregnancy loss prior to 24 weeks were classified as miscarriages and those occurring ≥24 weeks as stillbirths. The legal definition of stillbirth in England and Wales is a child that has issued forth from its mother after the 24th week of pregnancy and which did not at any time after being completely expelled from its mother breathe or show any other signs of life [26]. Patients’ electronic medical records were fully reviewed during data collection, including genetic results from invasive procedures and ultrasound findings during pregnancy. Additionally, newborns’ physical examination at discharge was reviewed and medical notes from those babies with suspected anomalies were examined in detail. We excluded pregnancies with aneuploidies or major fetal abnormalities diagnosed either prenatally or in the neonatal period. Women gave written informed consent to take part in the study, which was approved by the NHS Research Ethics Committee.

*Statistical analysis:* Data were expressed as mean (standard deviation) for continuous variables and n (%) for categorical variables. A Students *t*-test and an χ^2^-square test or Fisher’s exact test were used for comparing outcome groups for continuous and categorical data, respectively. A univariable logistic regression analysis was performed to examine the association between maternal race and stillbirth using White race as the reference. We used White race as the reference for three reasons: first, it represents the majority of our population, second, it allows comparisons with most other studies and pooling of the results, and third, it is the group with the lowest risk for the outcomes studied. Multiple logistic regression analysis with manual backward elimination was performed for stillbirth using race, age, weight, height, body mass index, mode of conception, smoking, history of chronic hypertension, diabetes mellitus and APS or SLE and obstetric history. The latter was subdivided into the following groups: nulliparous with no previous miscarriages, nulliparous with previous miscarriage at <16 weeks’ gestation, nulliparous with previous miscarriage at 16 + 0 to 23 + 6 weeks’ gestation, parous with no previous miscarriages or stillbirths, parous with previous miscarriage at <16 weeks’ gestation, parous with previous miscarriage at 16 + 0 to 23 + 6 weeks’ gestation, and parous with previous stillbirth. Before performing the multiple regression analysis, continuous variables were centered by subtracting the median from each measured value (67 from maternal weight in kg, 1.65 from maternal height in meters and 30 from maternal age in years). The statistical software R version 4.1.2 was used for data analysis [27].

### 2.2. Systematic Review and Meta-Analysis

Searches were carried out on the Ovid Medline, Embase, The Cochrane Library, Cinahl and Emcare databases identifying studies reporting on maternal race and stillbirth. The search was carried out on 10 August 2021 with no restriction for starting date but was restricted to English language records only; the initial search was updated with autoalerts in Medline to the end of March 2022. A list of relevant citations was generated from these databases using the search strategies given in the Methods section in the Supplementary Materials. This review was registered in the PROSPERO international database for systematic reviews (reference: CRD42021267548).

The abstracts of citations were examined by A.A. and D.A.N. to identify all potentially relevant articles, which were then examined in full-text form. Reference lists of relevant original and review articles were hand-searched for additional reports. Agreement about potential relevance was reached by consensus and by consultation with a third reviewer (K.H.N.). The inclusion criteria were peer-reviewed studies reporting on stillbirth in singleton pregnancies according to the race of women so that the rate of stillbirth could be compared in Black and South and East Asian women to the rate in White women. We excluded studies in which the earlier gestational age for definition of stillbirth was more than 24 weeks. We also excluded twin pregnancies, case-control studies and review articles or guidelines.

Data were obtained from each included study identified by the systematic review and documented in contingency tables. We extracted the necessary data to calculate the incidence of stillbirth in White, Black, South Asian and East Asian women. We also extracted the reported relative risk (RR) or odds ratio (OR) and 95% confidence intervals (CIs) from each study whenever possible. Finally, we extracted separate RR estimates with different degrees of confounder adjustment for the following prespecified conventional risk factors (age, weight and height or body mass index, smoking status and parity), where available. We conducted two meta-analyses: firstly, we used raw data to adjust random effect models for meta-analyses using the inverse variance method for pooling and DerSimonian-Laird to estimate the between-study variance (I^2^) and, secondly, we used adjusted ORs to also adjust the random effect model for meta-analysis with inverse variance for pooling but, in this case, restricted maximum-likelihood estimator (REML) for the between-study variance estimation was used. REML is a variation of the maximum likelihood (ML) used to correct the negative bias associated to the ML. It uses the Fisher scoring algorithm to iteratively search the value for which the change in τ^2^ estimate is smaller than 10^−5^ from one iteration to the next [28]. The pooled RR and/or pooled OR with 95% CIs were estimated for race as a predictor for stillbirth by a random effects model that considers both within-and between-study variation when using the adjusted analysis reported in the studies [29]. Statistical heterogeneity among studies was evaluated using the I^2^, τ^2^ statistics and the *p* value of the Chi-Square test of Q [30]. I^2^ is the fraction of variance across studies that is due to heterogeneity and not due to chance. A large value of I^2^ is interpreted as meaning that the effect size varies substantively across studies (>75 would represent considerable heterogeneity while less than 50 is generally considered low to intermediate heterogeneity). The I^2^ value must be interpreted together with the *p* value. Finally, the I^2^ statistics, which are used to estimate the prediction intervals, is a measure of the extent of variation, or heterogeneity, among the intervention effects observed in different studies [31]. The prediction interval is an index of dispersion that represents how widely the effect size varies across studies and, therefore, it is a property of the population, not the sample. This means that, unlike the confidence interval, which becomes smaller when the number of included studies increases, the true prediction interval stays constant regardless of how many studies we include in the analysis and only the estimate of the prediction interval will change as we add information [32].

Publication bias was assessed by plotting the RR estimate against precision (funnel plots) when the minimum number of included studies was 10 [33]. A funnel plot is a scatter plot of individual studies, their precision, and results. Each dot represents a study and, in the absence of publication bias, their distribution should resemble a pyramid or inverted funnel where one would expect to see an even scattering of trials on either side of this true underlying effect. On the contrary, when there is publication bias, an asymmetry in the scatter of smaller studies (those located at the bottom of the pyramid) is expected [33,34].

Risk of bias assessment was made with the quality in prognostic studies (QUIPS) tool [35] presented and adjusted for this review. The following six domains were used: representativeness of the study population, adequateness of the follow-up period and attrition, the appropriateness of race classification, the appropriateness of the definition of the outcome (stillbirth), and the adequateness of statistical analysis and reporting. Each element was classified as having a low, moderate or high risk of bias. An overall risk of bias for a study was graded as high if two of the domains were assessed as having a high risk of bias or four of the domains were assessed as having moderate risk of bias. The overall risk of bias was graded as moderate if three of the domains were assessed as having moderate risk of bias, or one domain was at high risk of bias and one was at moderate risk. Finally, the overall judgement for the study was low risk of bias if all the domains within a study were graded as low risk of bias, or less than three were moderate and none was high.

Statistical software R version 4.1.2 (The R Project for Statistical Computing, Vienna, Austira) was used in all analyses, packages meta and metafor were used for the meta-analysis and package car to clean the data [27,36,37].

## 3. Results

### 3.1. Fetal Medicine Foundation Study

In the FMF study there were 168,966 singleton pregnancies with a live fetus at 11 + 0 to 13 + 6 weeks without major abnormalities that delivered at ≥24 weeks of gestation; they included 601 (0.35%) stillbirths. In addition, there were 5406 (3.1% of the total) pregnancies that were not included in the study because there were no or incomplete data on pregnancy outcome.

The characteristics of the study population are summarized in Table 1. The incidence of stillbirth in Black women was higher than in White women; the incidence in South and East Asian women was not statistically significantly different that in White women. In Black compared to White women, there was a higher weight, a higher proportion of multiparous women, a higher incidence of chronic hypertension, type 2 diabetes mellitus, PE and SGA in a previous pregnancy as well as history of previous stillbirth and miscarriage, and a lower incidence of smoking and conception using assisted reproductive technologies. In South Asian women compared to White women, there was a higher maternal age, a higher proportion of multiparous women, and a higher incidence of chronic hypertension, type 2 diabetes mellitus, SLE/APS, conception using assisted reproductive technologies and SGA in a previous pregnancy; there was a lower weight, height, a lower incidence of type 1 diabetes mellitus, smoking during pregnancy and history of previous miscarriage at <16 weeks in nulliparous women. In East Asian women compared to white women, there was a lower weight, height, a lower proportion of multiparous women and a lower incidence of type 1 diabetes mellitus, smoking during pregnancy, previous PE and history of miscarriage at <16 weeks in multiparous women; there was a higher maternal age and a higher incidence of diabetes mellitus type 2 and previous SGA.

Table 2 reports the results of univariable and multiple logistic regression analysis demonstrating the association of maternal race with stillbirth. The analysis demonstrated that, first, Black compared with White women had significantly higher rates of stillbirth; second, South and East Asian women compared with White women had no significantly different rates of stillbirth; third, the odds ratio for stillbirth in Black compared with White women, after adjustment for elements of maternal characteristics and medical history, was 2.36 (95% CI 1.96, 2.84); and fourth, the results of multiple logistic regression analysis demonstrated that in addition to Black race, increased risk for stillbirth was provided by increasing maternal body mass index, conception after use of ovulation drugs, cigarette smoking, diabetes mellitus type 1, chronic hypertension, and previous pregnancy affected by stillbirth; the risk was reduced in parous women without previous miscarriage or stillbirth and in parous women with miscarriage <16 weeks’ gestation.

### 3.2. Systematic Review and Meta-Analysis

**Figure 1:**
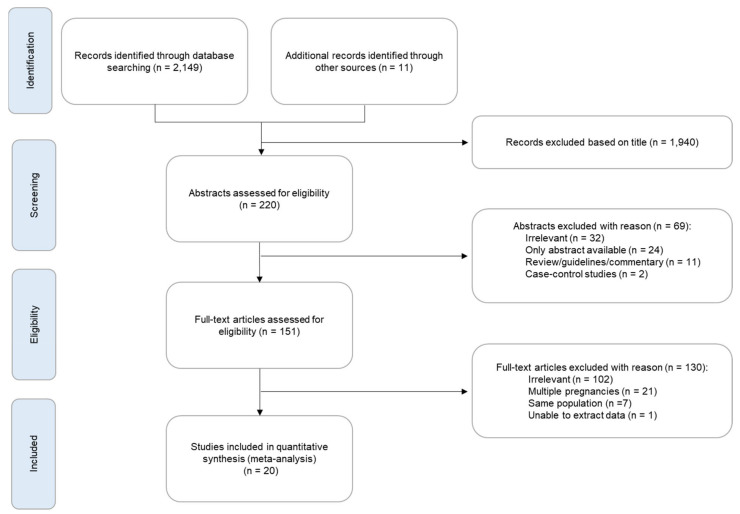
Flow chart for the systematic review.

The search identified 2160 potentially relevant studies, but 2140 were excluded because they were non-relevant articles, abstracts or letters, case-control studies, review articles, guidelines, studies providing data on a mixture of singleton and twin pregnancies where it was not possible to distinguish between the two, and studies on parts of the same population (Figure 1). In total, only 20 peer-reviewed papers were considered to be relevant and their data were combined with those of the FMF study for the meta-analysis [2,3,4,5,6,7,8,9,10,11,12,13,14,15,16,17,18,19,20,38]. In all 21 included studies, the populations were unselected singleton pregnancies. Definition of stillbirths varied between the studies: 11 studies defined stillbirth as pregnancy loss occurring ≥20 weeks [2,5,6,7,8,9,11,15,16,17,20], two studies as loss ≥22 weeks [4,13], one study as loss ≥23 weeks [10], and seven as loss ≥24 weeks [3,12,14,18,19,21]. All 21 studies reported on stillbirth in White and Black women, five on South Asian women and three on East Asian women.

The methodological quality of the selected studies, assessed the with the QUIPS tool [36], is illustrated in Figure S1. Only four of the 20 previous studies were considered to be at low-risk of bias, five were at moderate-risk of bias and 11 were at high-risk of bias. The main problem with most studies is that they did not adjust for confounders.

**Figure 2:**
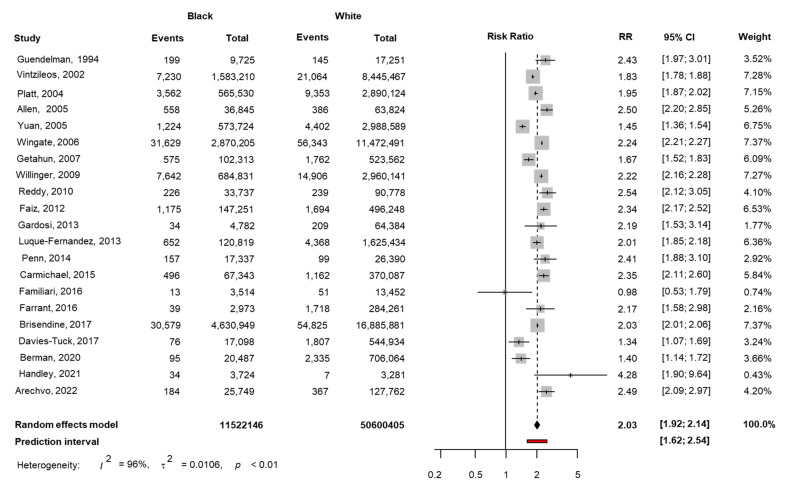
Forest plots of risk ratio for stillbirth in Black women compared with White women with 95% confidence intervals (CI) and weighted pooled summary statistics using a bivariate random-effects model.

The prevalence of stillbirth for each study, weighted pooled data and heterogeneity between studies are provided in Figure 2, Figures S2 and S3. In the meta-analysis of the combined data from 20 studies in the literature and the FMF study, the RR for stillbirth in Black compared with White women was 2.01 (95% CI 1.91, 2.12), but the heterogeneity of the studies was 96% (Figure 2). Publication bias was graphically assessed in Figure S4; the funnel plot showed no obvious asymmetry, but small studies are likely not published.

In the combined data from five studies, the incidence of stillbirth in South Asian compared with White women was significantly higher (RR 1.25, 95% CI 1.02, 1.54); the heterogeneity between studies was 80% (Figure S2). In the combined data from three studies, the incidence of stillbirth in East Asian compared with White women was not significantly different (RR 0.86, 95% CI 0.55, 1.34); the heterogeneity between studies was 75% (Figure S3).

**Figure 3:**
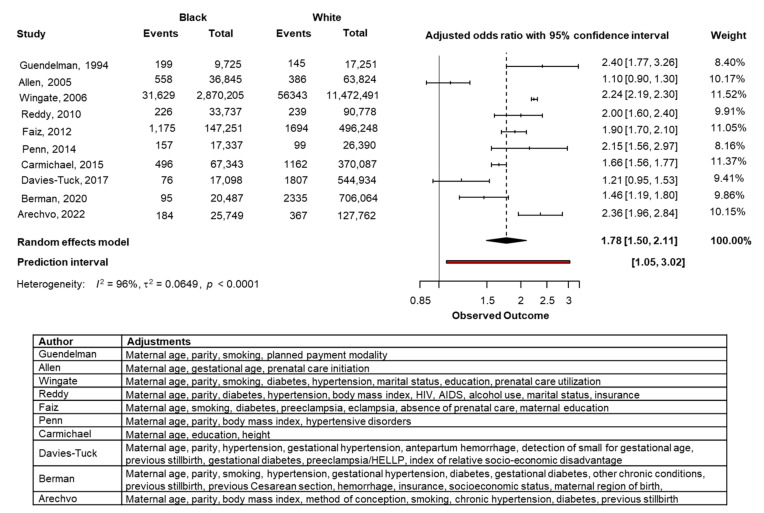
Forest plots of odds ratio for stillbirth in Black women compared with White women with 95% confidence intervals (CI) and pooled summary statistics using a bivariate random-effects model.

Only ten previous studies provided adjusted ORs and the results of the combined meta-analysis from these studies and our study are shown in Figure 3 and Figure S4. In Black compared with White women, the adjusted OR for stillbirth was 1.78 (95% CI 1.50, 2.11) and in South Asian compared with White women, the adjusted OR for stillbirth was 1.56 (95% CI 1.10, 2.21). There were no studies providing adjusted ORs in women of East Asian race.

## 4. Discussion

### 4.1. Main Findings

There are two main findings from the large FMF prospective study in women with singleton pregnancies living in England. First, a multiple logistic regression analysis demonstrated that increased risk for stillbirth was provided by the Black race, increasing maternal body mass index, conception after use of ovulation drugs, cigarette smoking, diabetes mellitus type 1, chronic hypertension, and previous pregnancy affected by stillbirth; the risk was reduced in parous women without previous miscarriage or stillbirth and in parous women with miscarriage <16 weeks’ gestation. These findings are consistent with those of our previous study of 113,415 singleton pregnancies [21]. Second, in Black women compared with White women, after adjustment for elements of maternal characteristics and medical history, there was a 2.4-fold higher risk of stillbirths; in South and East Asian women the rate of stillbirth was not significantly different from that in white women.

The literature search identified only 20 studies that provided data on the incidence of stillbirth in some of the racial groups as defined by the FMF study. In the assessment of the quality of the included studies, only four were considered to be at low risk of bias. In the meta-analysis of data from previous studies combined with those of the FMF study, the unadjusted risk of stillbirth in Black women was twofold higher and in South Asian women the risk was 1.3-fold higher than in White women; in East Asian women the risk was not significantly different to that in White women. In the meta-analysis of a small number of previous studies that provided adjusted ORs, albeit with adjustment for very few relevant maternal characteristics, combined with our data, risk of stillbirth in Black women was 1.8-fold higher and in South Asian women the risk was 1.6-fold higher than in White women; there were no studies providing adjusted ORs in women of East Asian race.

### 4.2. Interpretation of Results and Implications for Clinical Practice

Development of models for prediction of stillbirth and assessment of the contribution of race necessitates, first, data obtained from large prospective observational studies with accurate recording of maternal demographic characteristics and medical history and the appropriate infrastructure for obtaining the necessary outcome measures, and second, use of multiple logistic regression analysis to define the independent contribution of each risk factor. The data from the FMF study fulfil these criteria, demonstrating how the several elements from the maternal history contribute to stillbirth. In defining the specific contribution of one risk factor, such as Black race, it is essential that all other factors are taken into account.

In a previous study involving 131,514 of the pregnancies included in the current study, we reported that 92% of stillbirths were antepartum and 8% were intrapartum. About 60% of the antepartum stillbirths were thought to be due to impaired placentation, because the fetuses were small-for- gestational-age and/or the women had developed pre-eclampsia, and the other 40% were due to other causes or were unexplained [39]. Multivariable regression analysis showed that significant contribution to increased risk of impaired placentation-related stillbirths was provided by Black race, increasing body mass index, cigarette smoking, chronic hypertension, and previous pregnancy complicated by stillbirth, or preeclampsia or birth of small-for-gestational-age neonate. Black women had a threefold higher risk than White women, but the risk for South and East Asian women was not significantly different from that in White women. In the current study we did not attempt to categorize stillbirths according to the likely underlying cause because we wanted to compare our results to those reported in the literature, and previous studies did not provide comparable data.

The systematic review and meta-analysis has highlighted the weakness of such an approach in defining the contribution of one specific risk factor such as race. Although the combined number of patients arising from such studies can be very large, the heterogeneity between individual studies and the lack or minimal adjustment for confounders produces results that cannot be used for accurate prediction of the outcome under investigation.

### 4.3. Strengths and Limitations

The strengths of the FMF study are the prospective examination of a large multiracial population of pregnant women with singleton pregnancies attending for routine pregnancy care, accurate recording of maternal and pregnancy characteristics and medical history to identify known risk factors for stillbirth and the use of multiple regression analysis to identify independent predictors of stillbirth and define the relative predictive value of each factor. A limitation of the study is that race was classified into five broad categories and it is likely that there would be variations in outcome in subgroups within each category, such as different regions of Africa and between African and Caribbean women classified as Black. Additionally, we did not record data on the social determinants of health or index of multiple deprivation. However, our objective was to examine the relative incidence of stillbirth in the different racial groups rather than examine whether the origin of such differences was genetic or environmental.

The main limitations of the study relate to the findings of the systematic review of the literature and meta-analysis due to the high clinical and statistical heterogeneity found between the various included studies. For example, 20 studies provided data on the comparison of the incidence of stillbirth between Black and White women and although in most the incidence in Black women was higher, the heterogeneity between studies was 96%; furthermore, less than half of the studies reported adjusted ORs and adjustments were made for very few of the maternal characteristics. Similarly, there were only four studies reporting on South Asian women by comparison with White women and only three of these studies reported adjusted ORs. There were only two studies on East Asian women in comparison with White women and none of these studies reported adjusted ORs. Consequently, although the combined data included more than 11 million Black women and more than 50 million white women, the meta-analysis does not provide useful information on the true contribution of Black race to the prediction of stillbirth because of the heterogeneity between studies and the lack of adjustment for confounders in most of the studies; the same is true for women of South Asian and East Asian race.

## 5. Conclusions

The risk of stillbirth in Black women, after adjustment for confounders in maternal characteristics and medical history, is about twofold higher than in White women. The risk may also be increased, but to a lesser extent, in South Asian women. The study has highlighted that accurate assessment of the contribution of different racial groups to the prediction of stillbirth necessitates prospective examination of pregnancies and appropriate adjustment for confounders rather than meta-analyses of heterogeneous studies with no or minimal adjustment for confounders. A limitation of the FMF study was that we did not have data and therefore did not adjust for sociodemographic characteristics.

## Figures and Tables

**Table 1:** Demographic and pregnancy characteristics of the study population.

Characteristic	White (N = 127,762)	Black (N = 25,749)	South Asian (N = 7834)	East Asian (N = 3218)	Mixed (N = 4403)
Stillbirth	367 (0.287)	184 (0.715) *	25 (0.319)	11 (0.342)	14 (0.318)
Age (years)	30.8 (5.78)	30.7 (5.96) *	31.5 (4.99) *	32.4 (5.23) *	30.0 (6.07) *
Height (cm)	165 (6.51)	165 (6.42) *	159 (6.19) *	160 (5.96) *	164 (6.84) *
Weight (kg)	70.1 (15.4)	75.8 (16.8) *	63.3 (12.6) *	58.7 (10.2) *	69.5 (15.9) *
Conception					
Natural	122,760 (96.1)	25,314 (98.3) *	7477 (95.4) *	3079 (95.7)	4287 (97.4) *
In vitro fertilisation	3642 (2.85)	265 (1.03) *	273 (3.48) *	110 (3.42)	93 (2.11) *
Ovulation drugs	1360 (1.06)	170 (0.660) *	84 (1.07)	29 (0.901)	23 (0.522) *
Smoking	13,855 (10.8)	1020 (3.96) *	90 (1.15) *	44 (1.37) *	432 (9.81) *
Chronic hypertension	1182 (0.925)	891 (3.46) *	101 (1.29) *	20 (0.622)	44 (0.999)
Diabetes mellitus Type 1	622 (0.487)	62 (0.241) *	21 (0.268) *	5 (0.155) *	15 (0.341)
Diabetes mellitus Type 2	541 (0.423)	519 (2.02) *	200 (2.55) *	37 (1.15) *	33 (0.749) *
SLE/APS	262 (0.205)	78 (0.303) *	27 (0.345) *	4 (0.124)	8 (0.182)
Nulliparous	61,899 (48.4)	9653 (37.5) *	3634 (46.4) *	1622 (50.4) *	2106 (47.8)
Previous miscarriage <16 weeks	10,346 (8.10)	1731 (6.72) *	549 (7.01) *	261 (8.11)	380 (8.63)
Previous miscarriage 16–23 weeks	266 (0.208)	241 (0.936) *	25 (0.319)	7 (0.218)	20 (0.454) *
Parous	65,863 (51.6)	16,096 (62.5) *	4200 (53.6) *	1596 (49.6) *	2297 (52.2)
Previous PE	3829 (3.00)	1208 (4.69) *	229 (2.92)	46 (1.43) *	113 (2.57)
Previous SGA	7530 (5.89)	2830 (11.0) *	1032 (13.2) *	291 (9.04) *	390 (8.86) *
Previous stillbirth	862 (0.675)	446 (1.73) *	65 (0.830)	21 (0.653)	39 (0.886)
Previous miscarriage <16 weeks	17,767 (13.9)	4003 (15.5) *	990 (12.6) *	358 (11.1) *	633 (14.4)
Previous miscarriage 16–23 weeks	694 (0.543)	437 (1.70) *	52 (0.664)	11 (0.342)	38 (0.863) *

Values are given as median (interquartile range) or number (%); PE, preeclampsia; SLE, systemic lupus erythematosus; APS, antiphospholipid syndrome; SGA, small for gestational age <10th percentile. * This indicates significant difference from the finding in the White race.

**Table 2:** Odds ratios obtained from logistic regression analysis demonstrating association of maternal race with stillbirth.

Predictors	Univariate			Multivariate		
	OR	95% CI	*p*-Value	OR	95% CI	*p*-Value
Maternal age (years)	0.78	0.71–0.87	<0.001	0.85	0.77–0.94	0.001
Maternal age (years)^2^	1.00	1.00–1.01	<0.001	1.00	1.00–1.00	0.001
Body mass index (kg/m^2^)	1.05	1.04–1.07	<0.001	1.04	1.03–1.05	<0.001
Race						
White (reference)						
Black	2.50	2.09–2.98	<0.001	2.36	1.96–2.84	<0.001
East Asian	1.19	0.61–2.06	0.569	1.46	0.75–2.55	0.216
South Asian	1.11	0.72–1.63	0.610	1.26	0.82–1.86	0.262
Mixed	1.11	0.62–1.82	0.709	1.12	0.62–1.84	0.681
Conception by in vitro fertilization	1.17	0.73–1.87	0.512	1.14	0.67–1.82	0.598
Conception by ovulation drugs	1.89	1.04–3.43	0.038	1.96	1.01–3.40	0.028
Smoking	1.69	1.33–2.11	<0.001	1.92	1.50–2.43	<0.001
Diabetes Type 1	4.02	2.00–7.14	<0.001	3.90	1.93–6.95	<0.001
Diabetes Type 2	2.84	1.55–4.72	<0.001	1.47	0.79–2.50	0.187
Chronic hypertension	3.67	2.45–5.27	<0.001	2.04	1.34–3.01	0.001
Previous obstetric history						
Nulliparous (reference)						
Nulliparous-previous miscarriage <16 weeks	0.95	0.69–1.28	0.742	0.87	0.63–1.17	0.369
Nulliparous-previous miscarriage 16–23 weeks	2.36	0.84–5.16	0.058	1.36	0.48–3.00	0.505
Parous-no previous miscarriage/stillbirth	0.80	0.67–0.97	0.023	0.70	0.57–0.85	<0.001
Parous-previous miscarriage <16 weeks	0.87	0.67–1.12	0.292	0.71	0.54–0.92	0.012
Parous-previous miscarriage 16–23 weeks	0.85	0.26–2.00	0.750	0.49	0.15–1.18	0.166
Parous-previous stillbirth	4.08	2.55–6.17	<0.001	2.55	1.58–3.92	<0.001

To introduce a quadratic term in the model that considers the non-linear relationship between age and risk of stillbirth, maternal age was included plainly and squared (y = a + bx + cx^2^). Our data was distributed in this way, showing that both younger and older women have an increased risk of stillbirth.

## Data Availability

Research data are not shared.

## Supplementary Materials

The following supporting information can be downloaded at: https://www.mdpi.com/article/10.3390/jcm11123452/s1, Figure S1: Forest plots of risk ratio for stillbirth in women of South Asian race compared to white women with 95% confidence intervals (CI) and weighted pooled summary statistics using bivariate random-effects model; Figure S2: Forest plots of risk ratio for stillbirth in women of East Asian race compared to white women with 95% confidence intervals (CI) and weighted pooled summary statistics using bivariate random-effects model; Figure S3: Funnel plots demonstrating assessment of publication bias of studies reporting on the incidence of stillbirth in women of black and white race. Each dot represents a study; the y-axis represents study precision (standard error) derived from the number of experimental subjects and the x-axis shows the study’s result (risk ratio); Figure S4: Forest plots of odds ratio for stillbirth in women of South Asian race compared to white women with 95% confidence intervals (CI) and pooled summary statistics using bivariate random-effects model.
