# Supplementary material for: Maternal Race and Stillbirth: Cohort Study and Systematic Review with Meta-Analysis
Source: J Clin Med. 2022 Jun 15;11(12):3452. doi: 10.3390/jcm11123452 (PMC9224577; doi:10.3390/jcm11123452)
Supplement: Supplementary file 1 [file jcm-11-03452-s001.zip › jcm-1741161-supplementary.pdf]

Figure S1. Forest plots of risk ratio for stillbirth in women of South Asian race compared to white women with 95% confidence intervals (CI) and weighted pooled summary statistics using bivariate random-effects model.

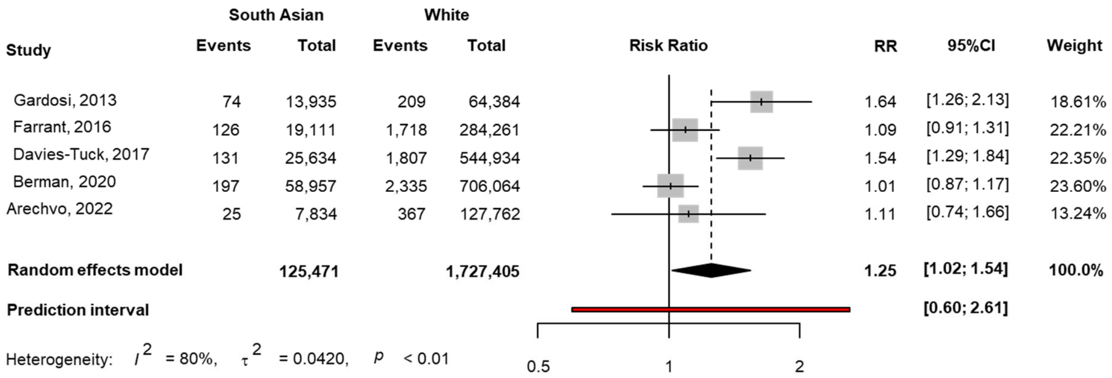

Figure S2. Forest plots of risk ratio for stillbirth in women of East Asian race compared to white women with 95% confidence intervals (CI) and weighted pooled summary statistics using bivariate random-effects model.

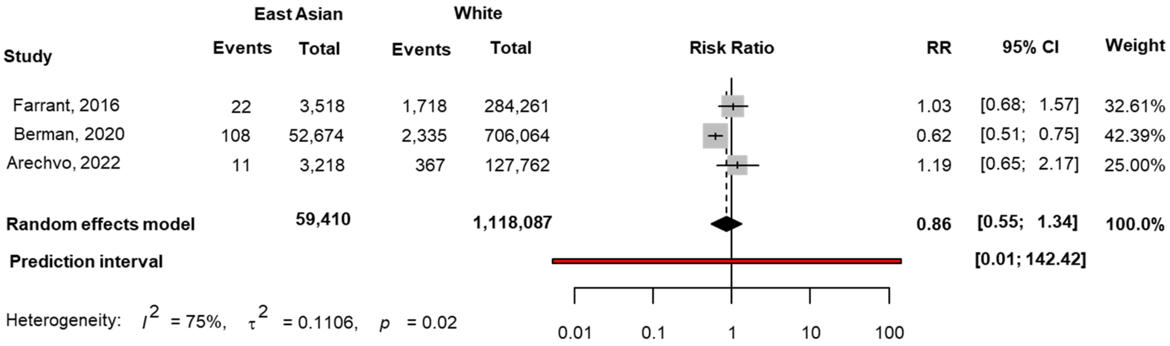

Figure S3. Funnel plots demonstrating assessment of publication bias of studies reporting on the incidence of stillbirth in women of black and white race. Each dot represents a study; the y-axis represents study precision (standard error) derived from the number of experimental subjects and the x-axis shows the study's result (risk ratio).

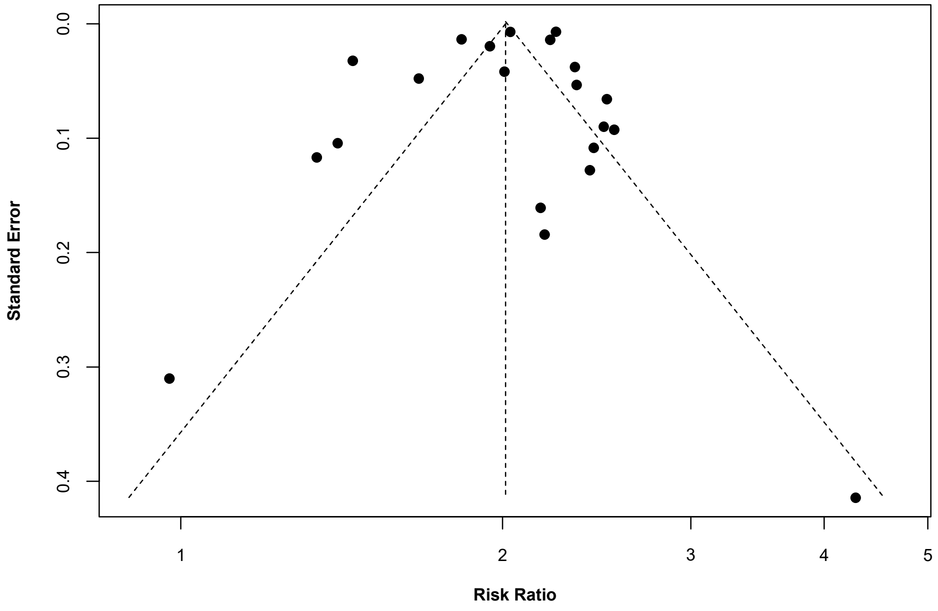

Figure S4. Forest plots of odds ratio for stillbirth in women of South Asian race compared to white women with 95% confidence intervals (CI) and pooled summary statistics using bivariate random-effects model.

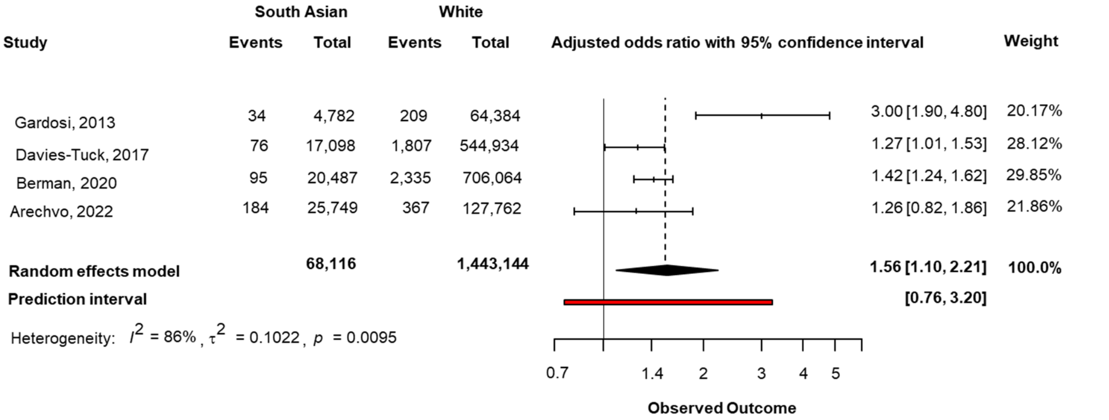

Author	Adjustments
Gardosi	Maternal age, parity, body mass index
Davies-Tuck	Maternal age, parity, chronic hypertension, gestational hypertension, preeclampsia/HELLP, antepartum hemorrhage, small for gestational age fetus, previous stillbirth, gestational diabetes, index of relative socio-economic disadvantage
Berman	Maternal age, parity, smoking, chronic hypertension, diabetes, other chronic conditions, gestational hypertension, gestational diabetes, previous stillbirth, previous Cesarean section, hemorrhage, insurance, socioeconomic status, maternal region of birth
Arechvo	Maternal age, body mass index, parity, method of conception, smoking, chronic hypertension, diabetes, previous stillbirth

Methods
Search strategies

Ovid MEDLINE(R) ALL <1946 to August 10, 2021>

1 exp african continental ancestry group/ or american native continental ancestry group/ or asian continental ancestry group/ or oceanic ancestry group/ or exp European Continental Ancestry Group/
2 (african-american* or caucasian* or black* or white* or african-caribbean* or afro-caribbean* or asian* or BAME or latin*).mp.
3 1 or 2
4 Stillbirth/
5 (still?born or still?birth or still-born or still-birth or f?etal death* or f?etal demise or intrauterine death* or intrauterine demise).ti,ab.
6 4 or 5
7 3 and 6
8 exp animals/ not humans.sh.
9 7 not 8
10 limit 9 to english language

Embase <1974 to 2021 Week 31>

1 exp african/ or exp black person/ or asian continental ancestry group/ or ancestry group/ or asian american/ or british asian/ or caucasian/ or hispanic/ or indigenous people/ or oceanic ancestry group/ or european/ or exp central european/ or exp eastern european/ or exp northern european/ or exp southern european/ or exp western european/
2 (african-american* or caucasian* or black* or white* or african-caribbean* or afro-caribbean* or asian* or BAME or latin* or hispanic*).mp.
3 1 or 2
4 stillbirth/
5 (still?born or still?birth or still-born or still-birth or f?etal death* or f?etal demise or intrauterine death* or intrauterine demise).ti,ab.
6 4 or 5
7 3 and 6
8 (exp animals/ or exp invertebrate/ or animal experiment/ or animal model/ or animal tissue/ or animal cell/ or nonhuman/) and (human/ or normal human/ or human cell/)
9 exp animals/ or exp invertebrate/ or animal experiment/ or animal model/ or animal tissue/ or animal cell/ or nonhuman/
10 9 not 8
11 7 not 10
12 limit 11 to english language
13 limit 12 to exclude medline records

Cinahl database 1981 – present

S1 MH "Ethnic Groups+"
S2 african-american* or caucasian* or black* or white* or african-caribbean* or afro-caribbean* or (east N1 asian*) or (south N1 asian*) or asian* or mixed race or BAME or latin* or hispanic*
S3 S1 OR S2
S4 MH "Perinatal Death"
S5 still?born or still?birth or still-born or still-birth or f?etal death* or f?etal demise or intrauterine death* or intrauterine demise
S6 S4 OR S5
S7 S3 AND S6
S8 MH Animals+
S9 MH (ANIMAL STUDIES)

S10 TI (ANIMAL MODEL*)
S11 S8 OR S9 OR S10
S12 MH (HUMAN)
S13 S11 not S12
S14 S7 not S13
S15 S7 not S13

Emcare 1995 – present

1 exp AFRICAN/ OR "AFRICAN AMERICAN"/ OR "AFRICAN BRAZILIAN"/
2 exp ASIAN/ OR "ASIAN AMERICAN"/ OR "ASIAN CONTINENTAL ANCESTRY GROUP"/
3 exp "OCEANIC ANCESTRY GROUP"/
4 exp EUROPEAN/
5 (african-american* OR caucasian* OR black* OR white* OR african-caribbean* OR afro-caribbean* OR (east ADJ1 asian*) OR (south ADJ1 asian*) OR asian* OR mixed race OR BAME OR latin* OR hispanic*).ti,ab
6 (1 OR 2 OR 3 OR 4 OR 5)
7 exp STILLBIRTH/ OR "STILLBORN BABY"/ OR "FETUS DEATH"/
8 (still?born OR still?birth OR still-born OR still-birth OR f?etal death* OR f?etal demise OR intrauterine death* OR intrauterine demise*).ti,ab
9 (7 OR 8)
10 (6 AND 9)
11 exp ANIMAL/
12 exp HUMAN/
13 11 not 12
14 10 not 13
15 ENGLISH LANGUAGE

Cochrane library

1 african-american* or caucasian* or black* or white* or african-caribbean* or afro-caribbean* or (east NEAR/1 asian*) or (south NEAR/1 asian*) or asian* or mixed race or BAME or latin*
2 still?born OR still?birth OR still-born OR still-birth OR f?etal death* OR f?etal demise OR intrauterine death* OR intrauterine demise*
3 1 and 2
